# Type, frequency and purpose of information used to inform public health policy and program decision-making

**DOI:** 10.1186/s12889-015-1581-0

**Published:** 2015-04-15

**Authors:** Pauline Zardo, Alex Collie

**Affiliations:** School of Population and Global Health, The University of Melbourne, Melbourne, Australia; Institute for Safety, Compensation and Recovery Research (ISCRR), Monash University, Melbourne, Australia; Department of Epidemiology and Preventive Medicine, Monash University, Melbourne, Australia

**Keywords:** Research, Use, Research translation, Policy, Government, Decision-making, Translation, Evidence, Public health

## Abstract

**Background:**

There is a growing demand for researchers to document the impact of research to demonstrate how it contributes to community outcomes. In the area of public health it is expected that increases in the use of research to inform policy and program development will lead to improved public health outcomes. To determine whether research has an impact on public health outcomes, we first need to assess to what extent research has been used and how it has been used. However, there are relatively few studies to date that have quantitatively measured the extent and purpose of use of research in public health policy environments. This study sought to quantitatively measure the frequency and purpose of use of research evidence in comparison to use of other information types in a specific public health policy environment, workplace and transport injury prevention and rehabilitation compensation.

**Methods:**

A survey was developed to measure the type, frequency and purpose of information used to inform policy and program decision-making.

**Results:**

Research evidence was the type of information used least frequently and internal data and reports was the information type used most frequently. Findings also revealed differences in use of research between and within the two government public health agencies studied. In particular the main focus of participants’ day-to-day role was associated with the type of information used. Research was used mostly for conceptual purposes. Interestingly, research was used for instrumental purposes more often than it was used for symbolic purposes, which is contrary to findings of previous research.

**Conclusions:**

These results have implications for the design and implementation of research translation interventions in the context within which the study was undertaken. In particular, they suggest that intervention will need to be targeted to the information needs of the different role groups within an organisation. The results can also be utilised as a baseline measure for intervention evaluations and assessments of research impact in this context.

## Background

Universities and research institutes are coming under increasing pressure from funders to demonstrate that research is utilised by industry and government and contributes positively to community outcomes [1,2]. Increasing use of research in public health decision-making is expected to increase the effectiveness and efficiency of policies and programs and lead to improved public health outcomes [3,4]. Whilst there has been much research identifying the barriers and facilitators to using research in public health policy environments and much effort invested in developing theoretical models to guide research translation processes, evidence of effective research translation interventions lags behind [5-7]. In other words, we know what the barriers to research use within public health policy are, but we don’t have enough valid evidence demonstrating the best ways to tackle them.

To evaluate the effectiveness of research translation interventions and assess the impact of increased research use on public health policy and program outcomes, effective measures of research use are needed [8,9]. Only a small number of existing published studies have quantitatively assessed how and to what extent research is used in policy environments [7,10]. Even fewer have quantitatively assessed research use in comparison to use of other information types [7,11-13]. In two of the largest and most cited published studies quantifying how and to what extent government policy decision-makers use research, one examined use of research in different decision-making stages [14]; the other measured policy decision-makers’ instrumental, conceptual and symbolic use of research in ‘day-to-day’ decision making processes [11].

Whilst these and other studies have made an important contribution to understanding how policy decision-makers use research, examining use of research evidence alone provides us with a limited understanding of research use [15]. For example, examining research use in isolation, as opposed to in comparison with use of other information types, makes it difficult to assess whether research use is high or low and in fact increasing or not. It is also well understood that research will only ever be one input amongst a range of inputs including politics, expert opinion, community opinion and government reports, that will inform policy decision-making [16-18]. Examining research in isolation also makes it difficult to identify how research evidence can complement, be integrated with or differentiated from other information types that are also used to inform policy and program decision-making.

There is also a need to explore how different types of decision-makers within a specific policy environment use research. Several studies of research use have looked at the difference between decision-makers working at different levels within an organisation or context. For example, studies have identified differences between policy decision-makers who work directly for Ministers compared to those who work within departments [19,20], and also between decision-makers in Executive, or Director level positions in a government department compared to those who work at lower levels [21-23]. Other studies have looked at differences between government and non-government public health organisations [24]. There are also many studies that fail to differentiate between different types of policy decision-makers.

There has been less focus on how the different focus of different policy decision-makers’ roles affects use of research. For example, how research use might differ within one organisation between those who focus on public health *policy* development compared to development and implementation of public health *programs*. In particular, there are a limited number of studies that have quantitatively assessed how research use differs across different types of decision-making roles within a single public health policy organisation. Studies that have shown differences suggest this a critical area to explore and address in studies of research use within specific public health policy organisations [25]. Such evidence can be used to target and tailor research translation strategies to the needs of different groups within public health policy organisations.

This study sought to measure the use of research, the frequency of use and the main purpose for use in comparison to use of other information types within two public health government agencies. This study also sought to examine whether there were differences in information use across different role levels and different role focus categories within the government agencies studied.

## Methods

### Study context

The two government public health agencies studied, WorkSafe Victoria and the Transport Accident Commission (referred to here on in as Agency 1 and Agency 2 respectively) are responsible for workplace and transport injury prevention and rehabilitation compensation in the Australian state of Victoria, which as at September 2012 had a population of over 5.5 million people [26].

The Agencies have a very similar mandate, but are structured differently and operate differently. Agency 1 has responsibility for both the Occupational Health and Safety (OHS) *Act, 2004* (VIC) and the *Accident Compensation Act, 1986* (VIC); which are primarily focused on workplace injury and illness prevention and return to work after injury or illness. Agency 1 is responsible for the enforcement of the OHS Act and related Regulations through workplace Inspectors, legal review and public prosecution. Agency 2 administers the *Transport Accident Act, 1986* which is focused on effective rehabilitation for those injured in a transport accident. Injury prevention policy and program development for this Agency is undertaken in partnership with the Victorian Police, VicRoads (the state government vehicle licensing and registration authority) and the Victorian Department of Justice [27]. Another key difference is the management of compensation claims. In Agency 1 management of claims is contracted out to private insurance agencies to conduct on their behalf, where Agency 2 employs claims managers within their Agency.

In 2009 these Agencies partnered with Monash University to establish a research Institute dedicated to workplace and transport injury prevention and rehabilitation compensation research. The aim of establishing this research Institute was to ensure that these Agencies would receive research that is relevant to their decision-making needs and actionable within their context. Ethics approval to survey these Agencies was received from Monash University Human Research Ethics Committee.

### Survey development

A quantitative survey was developed to quantify types, extent and purpose of use of research in comparison to use of other information types as well as measure individual level factors that affect use of research. Survey development was based on findings from a broad reading of the literature regarding the use of research in health policy decision-making environments, qualitative interviews with employees from Agencies 1 and 2, and Michie, Johnstone et al.’s domain framework [28] as well as questions from a survey developed by Campbell, Redman et al. [29]. At the time of survey development there were no ‘gold standard’ validated surveys appropriate for use with government policy and program decision-makers [24].

The Michie, Johnstone et al. domain framework (2005) is based on a systematic review of psychological and social-psychological theory relevant to behaviour change regarding the use of research. Twelve domains are comprised of related constructs and factors that affect research use. This framework outlines those domains that should be addressed by the development of related questions in interviews or surveys seeking to determine the capacity for research use. This framework was originally developed to assess clinicians’ use of research. However, the domains represent concepts found to affect policy decision-makers’ use of research, including skills, environmental context, resources etc. [30,31] and was therefore deemed relevant for use with policy decision-makers.

The survey was comprised of four main Parts. Part 1 measured use of information types, frequency of use of information and purpose of use of information. The results from this Part of the survey are the focus of analyses for this paper, results from Part 2 and 3 described below will be published in subsequent papers. Part 4 covered demographic questions related to individuals and to their organisation. Questions for all Parts of the survey were focused at the individual level. That is, questions were framed as follows: ‘how do you use…’, ‘what is your view…’, ‘in your day to day work…’; the emphasis is on the individual’s view and experience about their own behaviour, not that of others or the organisation as a whole.

All questions described below were closed ended and were compulsory in the survey. Some questions were followed with a non-compulsory qualitative question, which allowed participants to provide further information regarding their response. Participants were asked to consider their use of information in their day-to-day work within the last 12 months. Limiting the timeframe to the last 12 months can assist participants to more accurately recall their information use. Answers to all questions were self-reported and therefore the responses are participants’ perceptions and beliefs regarding their own use of information.

### Part 1

Part 1 included three main questions: ‘what types of information do you use’? ‘How often do you use the information types’? ‘For what purpose do you use the information types’? The categories of information type used in Part 1 (outlined below) were informed through review of relevant literature [18] and qualitative interviews undertaken with 33 employees from the two Agencies [32]. In the interviews participants were asked to describe the types of information they used to inform their work. Thematic analyses of the responses resulted in the development of information type categories, which were also reviewed and endorsed by project key contacts within the Agencies. The six information types and the definitions that were used throughout the survey are outlined below.

- Internal Data and Reports: Information, data and statistics collected, analysed and reported internally.
- Policy, Legislation and Legal Information: Acts and Regulations, or other policies and guidelines developed and/or administered by your organisation or by other government organisations, legal decisions, case law, industrial relations law, other legal documents and legal advice etc.
- Medical and Clinical Evidence: Medical certificates, doctors’ certificates, evidence provided in medical reports and hospital notes, any documentation provided by a GP, medical specialists, hospitals, pharmacies, etc.
- Experience, Expertise and Advice: Professional experience, expertise, advice and anecdote from people inside or external to your organisation; any information or advice you gain by asking questions and talking to your colleagues, your manager, stakeholders, technical specialists, experts, etc.
- Academic Research Evidence: Peer reviewed journal articles, reports of academic/scientific research, academic conference abstracts and papers.
- Information Collected Online: Any other information/evidence obtained from the Internet. Online information can include documents from your own organisation publicly available online, government reports, other organisations’ documents and websites, newsletters, any websites/web pages, etc. NOT information/evidence that fits into the other categories.

In Question 1, participants could select one or more information types from the list of six. The survey was filtered based on answers to Question 1; in subsequent questions each participant was only asked about the information types selected in Question 1. For example, if a participant only selected ‘Internal Data and Reports’ in Question 1, in Question 2 and 3 they would only be asked about the frequency and purpose for that one information type. This meant that the number of questions answered by each participant varied. Frequency of use of information was measured on a five point scale from ‘daily’ to ‘yearly’ for each information type and only one option could be selected.

The development of categories of purpose of use of information was informed by a reading of the literature on use of research evidence. Three distinct uses of research; instrumental, conceptual and symbolic, have been conceptualised and developed over time, based on earlier studies that examined how research is utilised [33,34]. This conceptualisation of research use has been widely adopted and utilised in the research translation literature [11,16,35,36].

Instrumental use of research refers to the direct, specific application of research findings in the development of policies and/or programs [37]. Conceptual use refers to the use of research to inform policy and program decision-makers’ understanding of an issue, often referred to as enlightenment, but may not lead to direct application in policy and program formulation [33,37]. Symbolic use refers to the use of research to support ideas, positions or actions that have already been taken or decided upon [37].

Participants were asked to rank their purpose for use of each information type they used, from ‘main reason for use’ to ‘least main reason for use’, based on the three types of research use. The three types of research use were described in the survey as follows: Instrumental use - To act on the information/evidence in specific and direct ways (documented use); Conceptual use - To inform, generally or indirectly, understanding of an issue (not documented, not for a specific purpose); Symbolic use - To support or argue for certain positions or plans of action (not documented, but for a specific purpose).

### Part 2

Part 2 of the survey was comprised of five main questions focused on sources of academic research evidence and included one question on sources of experience, expertise and advice and therefore was only answered by participants who selected these information types in Part 1. Some of the questions in this Part were based on the Campbell [29] study regarding policy-decision-makers’ ‘exchange’ or involvement with research and researchers through attending and arranging presentations and forums, commissioning research projects and involvement in research projects.

### Part 3

Part 3 was comprised of questions related to factors that can affect research use. Questions were developed in relation to the domains of the Michie & Johnstone [28] framework to ensure domains relevant to attitudes and behaviour regarding use of research were covered. There were also questions asking about barriers and facilitators to use of research, perceived relevance of research and preferences for the communication of research.

### Part 4

The last Part of the survey was comprised of demographic questions related to the individual and their role within their organisation and within government. Aside from questions about gender, age and education level, participants were asked to indicate which of the two Agencies they worked for; their role level; role focus; length of employment in their current role; in their organisation; and in government roles. Although the latter information categories may not be considered standard demographic measures, they will be referred to from here on in as ‘demographics’.

Role level categories included Senior Manager, Manager and Non-Manager which were identified through a review of organisational charts and informed by interviews. The role level categories were defined in the survey as follows: Senior Manager - I manage Managers; Manager - I manage Non-Managers; Non-manager - I do not manage people.

The role focus categories sought to identify the type of work that the respondent was mainly engaged in on a day-to-day basis. The categories and descriptions developed were based on qualitative interviews undertaken where people described their roles and these were approved by the project key contacts. The role focus categories and their definitions are described below.

- Program/Projects: My work mainly involves strategy and program and project planning, management, evaluation, and/or mainly program and project support, tasks supporting program and projects etc.
- Policy/Legal: My work mainly involves the development and/or review of policy, legislation or legal information.
- Operational: My work mainly involves the implementation or delivery of strategy, policy, programs or projects, or tasks that support policy, programs or project implementation and strategic operations.
- Administration/Assistance: My work mainly involves office and business administration, executive assistance or administrative assistance etc.

### Survey testing

A total of eight individuals including employees that previously worked within the Agencies and employees from areas that were not selected to be part of the study, piloted the survey. Piloting resulted in greater detail being developed for the descriptions of information types and also greater clarity and description of the survey purpose in the survey introduction and invitation email. The program Qualtrics was used to distribute the survey and collect responses

Face validity of the survey was confirmed via feedback from those who piloted the survey, the Agencies’ key contacts for this project as well as via review of the survey by the authors of this paper. The survey covered key concepts addressed in other quantitative surveys in this field [11,29] and expanded on these, as well as addressing key concepts outlined in the research translation literature [7,30]. This suggests that content validity is present; however review by field experts in further iterations of the survey is required.

Reliability was tested by measuring internal consistency of the ordinal items only, as options for testing categorical survey item reliability was limited [38]. The split half method was used to calculate Cronbach’s Alpha for 78 valid cases, representing 21% of all cases. Cronbach’s Alpha for the first random split half was 0.61 and the second 0.56. The Spearman-Brown coefficient for both equal and unequal halves was 0.52. The Guttman split half coefficient, a conservative measure of split half reliability, was 0.51. A Cronbach’s Alpha of 0.50 to 0.60 is considered an acceptable level of reliability for a survey which is in the early stages of development [38,39]. As this is the first version of this survey developed and tested, and due to lack of ‘gold standard’ existing surveys in this field, these are positive results. This survey will be subject to further analyses and revision prior to re-administration.

### Selection of participants

Potential participants were identified by a review of organisational charts provided by project key contacts. Areas that were involved in the development, implementation or evaluation of policies, programs and projects were selected and confirmed by the key contacts. All employees from the selected areas (N = 1278) were included in the participant pool.

### Recruitment

Potential participants were invited to participate via emails sent by the Heads of the selected areas on behalf of the research team. The lead author developed the email invitation, which included a link to the survey, Participant Information Sheet and a document including definitions of the included information types. Instructions for the survey invitation mail out were also developed which included instructions on blocking the forwarding of emails to ensure only the intended participant pool received the email invitation. The first author was copied in to all email invitations and attempts to forward the emails confirmed the instructions had been followed. Participants were offered a prize for completing the survey; all participants who fully completed the survey entered the draw. This was also explained in the email invitation. First round invitations were mailed out between 11 and 23 November 2012. Agency 2 sent reminder emails to all selected areas on 10 December 2012. The survey closed on 21 December 2012. Participation in the survey was self-selected.

### Analysis

Survey data was extracted from the Qualtrics program as a Statistical Package for the Social Sciences (SPSS) data file and analysed in SPSS version 20. The categorical data presented here were analysed using chi-square analyses. Calculations of confidence intervals for proportions were also conducted. Participants who indicated that the main focus of their role was ‘Administration and Assistance’ were excluded from the analysis, as they were not the targets of the study. Role focus was not identifiable in the review of organisational charts and it was found to be too difficult to exclude specific roles as part of the survey mail out.

## Results

Of the 1278 people that were invited to participate, 405 fully completed the survey (response rate = 31.7%). Thirty-three participants whose main role focus was not relevant to the analysis were excluded. The following results are based on analyses undertaken for the remaining 372 participants.

Pearson Chi-square was computed for all demographics (Agency; role level; role focus; age; gender; education level; years in Agency; years in current role; years in government roles) by information type. Only demographics with a significant difference in distributions for use of information were included in Table 1 below.

**Table 1:** **Use of information type in the last 12 months**

	**Total**	**Agency**			**Role level**				**Role focus**				**Education level**				**Years in Agency**				**Age group**			
		**1 N %**	**2 N %**	**χ** ^**2**^ **Sig level**	**S M N %**	**M N %**	**NM N %**	**χ** ^**2**^ **Sig level**	**P/P N %**	**P/L N %**	**O N %**	**χ** ^**2**^ **Sig level**	**HS/C N %**	**UG N %**	**PG N %**	**χ** ^**2**^ **Sig level**	**0-5 N %**	**6-15 N %**	**16+ N %**	**χ** ^**2 **^ **Sig level**	**18-35 N %**	**36-55 N %**	**56+ N %**	**χ** ^**2**^ ** Sig level**
**Total (row %)**	372 100.0	146 39.2	226 60.8	N/A	15 4.0	75 20.2	282 75.8	N/A	125 36.6	47 12.6	200 53.8	N/A	117 31.5	151 40.6	104 27.9	N/A	245 65.9	111 29.8	16 4.3	N/A	163 43.8	187 50.3	22 5.9	N/A
**Internal data & reports**	347 93.3	137 93.8	210 92.9	0.89	15 100.0	73 97.3	259 91.8	0.12	123 98.4	35 74.5	189 94.5	0.000	110 94.0	138 91.4	99 95.2	0.66	226 92.2	106 95.5	15 93.8	0.52	149 91.4	176 94.1	22 100.0	0.26
**Policy, legislation & legal info**	343 92.2	133 91.1	210 92.9	0.66	14 93.3	70 93.3	259 91.8	0.90	106 84.8	47 100.0	190 95.0	0.000	110 94.0	137 90.7	96 92.3	0.68	222 90.6	106 95.5	15 93.8	0.27	145 89.0	179 95.7	19 86.4	0.036
**Medical & clinical evidence**	228 61.3	63 43.2	165 73.0	0.000	9 60.0	50 66.7	169 59.9	0.56	40 32.0	29 61.7	159 79.5	0.000	92 78.6	90 59.6	46 44.2	0.000	144 58.8	73 65.8	11 68.8	0.37	98 60.1	117 62.6	13 59.1	0.87
**Experience expertise & advice**	330 88.7	131 89.7	199 88.1	0.74	15 100.0	67 89.3	248 87.9	0.35	105 84.0	42 89.4	183 91.5	0.11	102 87.2	134 88.7	94 90.4	0.78	221 90.2	95 85.6	14 87.5	0.44	139 85.3	170 90.9	21 95.5	0.15
**Academic research**	145 39.0	71 48.6	74 32.7	0.002	10 66.7	33 44.0	102 36.2	0.038	64 51.2	22 46.8	59 29.5	0.000	29 24.8	56 37.1	60 57.7	0.000	88 35.9	51 45.9	6 37.5	0.20	58 35.6	82 43.9	5 22.7	0.08
**Info online**	291 78.2	121 82.9	170 75.2	0.11	12 80.0	61 81.3	218 74.9	0.74	103 82.4	36 76.6	152 76.0	0.38	88 75.2	122 80.8	81 77.9	0.06	185 75.5	96 86.5	10 62.5	0.020	118 72.4	154 82.4	19 86.4	0.050

**Legend:** Agency 1 = WorkSafe, 2 = TAC; Role level SM = Senior Manager, M = Manager, NM = Non Manager; Role Type P/P = Programs and projects, P/L = Policy and legal, O = Operational; Education Level HS/C = High school and/or Certificate; UG = Undergraduate, PG = Postgraduate; χ^2^ Sig level = Pearson Chi Square significance level.

**Notes:** Significant with at least 80% of cells with expected frequency of 5 or more. All column percentages unless otherwise state.

Of the 372 participants, 250 (67.2%) were women, 121 (32.5%) men, and 1 (0.3%) person who identified as ‘other’. The majority of participants (N = 332; 89.2%) had been in their current role for 0-5 years. Thirty-seven participants (9.9%) had been in their current role for 6-15 years and three (0.8%) had been in their current role for 16 years or more. Most participants (170; 45.7%) had worked in government roles for 0-5 years, 146 (39.2%) had been in government roles for 6-15 years, and 56 (15.1%) had spent 16 years or more in a government role.

### Use of information

The analyses revealed a significant difference in use of medical and clinical evidence and academic research evidence across Agencies. Agency 2 participants used more medical and clinical evidence (χ^2^ [1, n = 372] = 33.3, p = 0.000) and Agency 1 participants used more academic research evidence (χ^2^ [1, n = 372] = 9.41, p = 0.002).

Examination of role level identified that there was only a difference between Senior Managers, Managers and Non-Managers for use of academic research evidence (χ^2^ [2, n = 372] =6.56, p = 0.038).

Analyses of role focus showed the greatest differentiation in use of information types. More participants from program and project roles used internal data and reports, closely followed by those in operational roles, compared to participants from policy and legal roles (χ^2^ [2, n = 372] = 32.23, p = 0.000). All participants from policy and legal roles used policy, legislation and legal information. More participants from operational roles used policy, legislation and legal information than those in program and project roles (χ^2^ [2, n = 372] = 15.68, p = 0.000). Whilst a significant difference was found, at least 74.5% of all role groups used internal data and reports and policy, legislation and legal information.

Medical and clinical evidence was mostly used by those in operational roles, followed by those in policy and legal roles. Program and project participants were less likely to use this type of information (χ^2^ [2, n = 372] = 73.16, p = 0.00).

Program and project participants were most likely to use academic research evidence, followed by those in policy and legal roles. Participants in operational roles were the least likely to use research evidence (χ^2^ [2, n = 372] = 16.62, p = 0.00)

Education level was associated with use of medical and clinical evidence and use of academic research evidence. Participants with high school certificate and diploma level education as well as those with undergraduate degrees used medical and clinical evidence more than those with postgraduate level education (χ2 [2, n = 372] = 27.77, p = 0.00). Use of research evidence showed the opposite pattern where those with postgraduate research evidence used research evidence more than those with undergraduate degrees. Those with high school certificate and diploma level education used research evidence the least (χ2 [2, n = 372] = 25.45, p = 0.000).

Years in Agency was only associated with the use of online information where those who had been employed in their Agency for 6-15 years used online information more than those who had been with the Agency for 1-5 years or 16 years or more (χ^2^ [2, n = 372] = 7.83, p = 0.020).

Age was associated with use of policy, legislation and legal information and online information. Participants in the 36-55 year age group showed greater use of policy, legislation and legal information (χ^2^ [2, n = 372] = 6.65, p = 0.036). Participants in the 56 plus age groups showed greater use of online information (χ^2^ [2, n = 372] = 5.98, p = 0.050). None of the demographics measured were associated with use of experience, expertise and advice.

### Frequency of use of information

**Figure 1:**
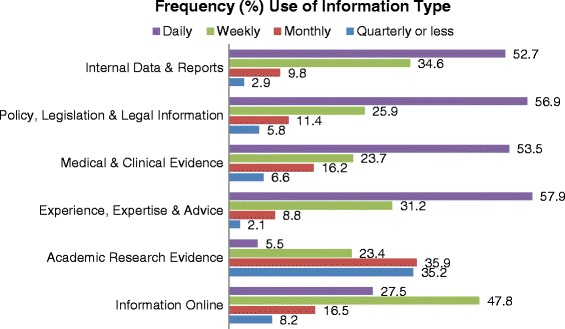
Frequency (%total) of use of information type in the last 12 months. Pearson Chi square = χ^2^ (15, n = 1684) = 368.03, p = 0.000. Note: Analysis for Figure 1 and Table 2 required restructuring of the data to long format, where each row represents one observation of use of evidence per row, rather than one case per row, resulting in N = 1684.

Frequency of use of information types is depicted in Figure 1 below.

The majority of information types were mostly used on a daily and weekly basis. Academic research was used much less frequently, most often used monthly, quarterly or less (χ^2^ [15, n = 1684] = 368.03, p = 0.000). Analysis undertaken on frequency of use of information (Figure 1) and purpose for use of information (Table 2) required restructuring of the data to long format, where each row represents one observation of use of evidence per row, rather than one case per row, resulting in N = 1684.

**Table 2:** **Main purpose for use of information**

**Main purpose for use of information type**	**Internal data and report N (%)**	**Policy, legislation and legal information N (%)**	**Medical/Clinical evidence N (%)**	**Experience, expertise and advice N (%)**	**Academic research evidence N (%)**	**Online information N (%)**
**Instrumental**	192 (53.3)	182 (53.1)	128 (56.1)	148 (44.8)	44 (30.3)	96 (33.0)
**Conceptual**	107 (30.8)	99 (28.9)	53 (23.2)	118 (35.8)	73 (50.3)	149 (51.2)
**Symbolic**	48 (13.8)	62 (18.1)	47 (20.6)	64 (19.4)	28 (19.3)	46 (15.8)
**Total use (1684)**	347	343	228	330	145	291

**Pearson Chi square =** χ^2^ (10, n = 1684) = 83.52, p = 0.000.

**Note:** % given is percentage of column total.

### Purpose for use of information

The main purpose or reason for use of information types is presented in Table 2 below.

Information type was associated with the main purpose for use of information (χ^2^ [10, n = 1684] = 83.52, p = 0.000). All information types, other than academic research evidence and online information, were mostly used for instrumental purposes, followed by conceptual then symbolic use. Academic research evidence and online information were mostly used conceptually, followed by instrumental and then symbolic use.

Confidence intervals (95%) for the proportions of main use of academic research evidence showed that research is significantly more likely to be used conceptually (65.77-80.23; ±7.23) than instrumentally or symbolically. Instrumental use (35.94-52.09; ±8.08) was more common than symbolic use, but only by a small margin (20.69-35.31; ±7.31).

## Discussion

Results from a quantitative survey undertaken with 372 participants have shown that use of information differed both across and within the two government public health Agencies studied. The information types most commonly used were those held or generated internally including internal data and reports, policy, legislation and legal information and experience, expertise and advice. In particular, these participants relied heavily on internal data and reports to inform their day-to-day decision-making. Analysis also revealed that academic research evidence is the information type used least often. Use of research evidence was mainly conceptual, meaning decision-makers mostly used research to inform their general understanding of a policy or program issue.

This study specifically sought to demonstrate information use ‘as is’ [40] in these Agencies and describe the range of information types that make a valid and important contribution to public health policy and program decision-making. Whilst it is increasingly recognised that many other information types inform policy and program development [10,40], there has been limited focus on identifying and understanding other information types in the literature on use of research. Oliver, Innvar et al.’s [10] systematic review found that most studies in the field fail to define evidence, and argued that identifying other ‘sources and types of information’ that inform decision-making is ‘a crucial step in describing and ultimately influencing the policy process’.

This study addresses these critical gaps in the literature and provides practical information that can be used by the Agencies studied and the research Institutes they fund. These Agencies have partnered with a University to fund a research Institute with the specific aim of producing research that is relevant to Agency decision-making needs and actionable in their context [41]. This suggests that they are seeking to increase the use of research in their policy and program development processes. These findings allow research use to be measured in relation to use of other information types in their organisations, which provides a more accurate and nuanced understanding of research use than is possible by measuring research in isolation of use of other information types.

The two Agencies differed in their use of academic research evidence and medical and clinical evidence (that provided by doctors and other clinical professionals). Differences in use of information types across Agencies can in part be explained by differences in the work undertaken by each Agency. Agency 2 undertakes claims management in-house, therefore they use more clinical and medical evidence as this is the key information type used to make decisions about whether a claim is eligible for compensation [42]. Agency 2 contract out this work, and as such use less of this type of information. Interestingly, decision-makers with different roles used different types of information to inform their work, suggesting that the type of work you do affects the types of information you use.

Previous studies have found that information is used differently across policy agencies [11,14]. The results of this study build on those findings and demonstrate that information is also used differently *within* policy agencies. In this context, participants from policy and program roles were the most likely to use academic research evidence and those in operational roles were the least likely. Whilst this survey was focused at the individual level, the differences aggregated at the organisational level supports calls for greater focus on the organisational context when designing research translation interventions [4,43-45].

An important finding is that program and project workers used research more than those in policy and legal roles, when often the literature in this field is directed at ‘policy’ decision-makers [10]. Whilst the difference was not large and results are based on a cross-sectional survey and findings may shift over time, it suggests it may be important to continue to explore factors that affect ‘program and project’ decision-makers’ use of research separately from ‘policy’ decision-makers’ use of research. Whilst some authors have intended to capture both types of roles with the term ‘policy-maker’, these findings suggest it is important to differentiate these roles.

In practice, the two groups make different types of decisions and results showed they use information differently. In this environment those in policy and legal roles develop, interpret and review legislation and policies, and provide broad guidance and advice as to what type of actions can be taken within the jurisdiction of the Agencies. The program and project workers are responsible for developing and implementing the specific public health programs and projects. The results showed that program and project workers relied more heavily on internal data and reports and policy and legal roles relied more on policy legislation and legal information and medical and clinical evidence. Further qualitative research in the Agencies studied and other public health focused government agencies would enhance understanding of the differences between policy and program focused roles and how and why information use differs across these roles.

Oliver, Innvar et al.’s [10] systematic review highlighted that much of the literature on use of research focuses on ‘policy making’ and fails to recognise that a significant aspect of the role of government is implementation of policy and programs, which is a different stage of the policy process [46,47]. The finding that research use differed across role focus groups *within* the Agencies supports the implication that information used to inform policy development and policy implementation is likely to differ [10]. These findings also suggest that interventions need to be targeted to the needs of different teams or divisions and roles within policy organisations. In particular, it indicates that information needs between policy and program and project decision-makers can differ and therefore communication and dissemination strategies need to be targeted and tailored to these needs.

While different role focus categories showed differences in use of a range of information types, role level only showed significant differences in use of academic research. Senior Managers were more likely to use research evidence than Managers and Non-Managers. This suggests that research evidence is currently more likely to be used at the strategic decision-making level, rather than at the individual policy, program and project level for which Managers and Non-Managers are responsible. Senior Managers reported higher education levels than Managers and Non-Managers, which may explain why their use of research was higher. Authority to implement change is an important facilitator to use of research [30] and Senior Managers are particularly well placed to influence change as they have significant authority over decision-making in the areas they manage [48]. These findings suggest a need for Senior Managers to drive use of research at the Manager level, as Managers are in a position to directly influence, or even require that their staff (Non-Managers) use research evidence in their work wherever relevant.

Whilst many participants did use research evidence, it was used less frequently than the other information types. The term ‘evidence-informed’ decision-making emerged in recognition of the fact that research evidence will only ever be one of several types of information and inputs that inform the development of public health policies and programs. This is because the policy and program development process is political and dynamic, driven by argumentation of ideas and values; it is not a linear, rational process based on the findings of research evidence [40,49,50]. Policy and program development has been described in theory and research as ‘interdependent and variable, an incremental, often messy process of ‘muddling’ through’ [51,52].

There are also many factors that affect policy and program decision-makers use of research. Systematic reviews conducted over that last decade consistently show that relevance of research to decision-makers need, timeliness of research produced in relation to decision-making timeframes, mutual trust and understanding, need for actionable recommendations and concise and engaging communications and capability and resources to support research use are key factors that affect research use [5,7,10,30,53]. In the Agencies studied five key factors have been found to predict research use: perceived relevance of research, skills for using research use, intention to use research, internal prompts for research use and Agency worked within [54]. In particular relevance and skills were strong predictors of research use. Decision-makers who viewed research as relevant to their work were 11 times more likely to be using research than those who though it was not relevant. Even those who found research ‘somewhat’ relevant were 5 times more likely to use research. Decision-makers who rated their skills for research use from high to very high and medium were respectively 4 and 2 times more likely to use research and those who rated their skill as low.

The findings reported in this paper can be utilised to better understand the range of information types that are used and considered relevant by and different types of decision-makers within the Agencies. It is important to recognise that there are many policy and program decisions that do not need to be informed by research evidence, and that different information types will be relevant to different types of policy and program issues, questions and decisions [33,40,55,56]. The systematic review by Oliver, Innvar (10) also indicated a need for more empirical research on the policy process. Research on the types of decisions made at different stages of the process, the teams and roles involved, and in particular if, when and how research evidence can support decision-making at different stages of the process is needed. Such evidence will be critical to assisting researchers to better understand the policy process and how research can and does fit within that process, which can in turn improve targeting of translation interventions.

Of the participants that did use research evidence in the Agencies studied, they mainly used it for conceptual purposes. Previous studies have also found that policy decision-makers’ main purpose for use of research is conceptual [11,36]. The findings of this study confirm previous findings, but extend upon these to demonstrate that other information types are more often used instrumentally than conceptually. This shows that research is used mostly to inform thinking and understanding around an issue, rather than used to directly inform specific action. This also supports the suggestion that in this context research tends to be used at a more strategic level, for example informing program and project prioritisation, or general understanding of priority issues, rather than to inform specific action at the project level such project targeting or implementation of evidence-based interventions.

Interestingly, there was more instrumental use of research evidence than symbolic use, which is contrary to previous research findings [11]. Previous studies have highlighted that similar organisations can have different approaches to and perspectives on research use, which may explain why these findings differed from other similar studies. However, there are a limited number of studies that have examined different types of research use and further studies are needed to understand differences in results. These results provide a particularly useful baseline measure for future impact assessments in the Agencies studied, as it will be possible to measure change in conceptual, instrumental and symbolic use of research in comparison to use of other information types [8].

Whilst many academics, and indeed research funders, might consider or expect that the best or most appropriate use of research is instrumental, it has been effectively argued that conceptual and symbolic use are also relevant, valuable uses of academic research [8,11,15,16]. Instrumental, conceptual and symbolic uses of research can be understood as complementary and each relevant and necessary at different stages of policy decision-making processes and for different policy issues [11,16].

This survey utilised to collect data for this study was designed for use in a specific public health policy context; however the results are consistent with similar studies that had larger sample sizes and were undertaken across a range of policy agencies. Moreover, the survey allowed for analysis of differences in use of information across different groups within organisations and between two closely related organisations. This suggests that this survey may be relevant for use in other public health policy settings if modified to address the specific context. For example, a modified version may include different information types and different role level and role focus categories.

## Conclusions

This study has confirmed findings of previous research and also revealed important new findings. In particular it makes an important contribution to the literature on policy decision-makers use of research, as research evidence was clearly defined and research use compared to use of other information types. This addresses a critical gap in the literature and helps to provide a more nuanced understanding of research use, and where it fits in the broader range of information types used use to inform decision-making. It has shown that research use is low in comparison to the use of other information types and is used differently across and within the two Agencies, showing that there are differences in use of research between program and project and policy decision-makers. This is critical because these groups are not often differentiated in similar studies.

Research was most often used conceptually, supporting previous research, but this study also revealed other information types were most often used for instrumental purposes. Contrary to the findings of previous research, it was found that instrumental use of research was more common than symbolic use. These findings indicate that research translation interventions designed for the workplace and transport injury prevention and rehabilitation compensation context need to be targeted and tailored to the different information needs of the different Agencies and role level and role focus categories within the Agencies. The results of this survey also provide baseline measures for an evaluation of research translation intervention effectiveness and assessments of research impact in the Agencies studied.

Further research on the types of information used and types of decision-makers involved in different stages or aspects of the policy and program development process is needed to better understand if, when, where and how use of research evidence can be an effective and valuable input to policy and program development. Such evidence could assist the design, implementation and evaluation of interventions seeking to increase research use as intervention effectiveness is another, related topic in the field that currently requires further research [6].

## References

[1] Lavis J, Ross S, McLeod C, Gildiner A (2003). Measuring the impact of health research. J Health Serv Res Policy.

[2] Donovan C (2011). State of the art in assessing research impact: introduction to a special issue. Res Evaluation.

[3] Task Force (2009). Scaling up research and learning for health systems: now is the time. Global Ministerial Forum on Research for Health 2008.

[4] Brownson RC, Fielding JE, Maylahn CM (2009). Evidence-Based Public Health: A Fundamental Concept for Public Health Practice. Annu Rev Public Health.

[5] Grimshaw J, Eccles M, Lavis J, Hill S, Squires J (2012). Knowledge translation of research findings. Implement Sci.

[6] Moore G, Redman S, Haines M, Todd A (2011). What works to increase the use of research in population health policy and programmes: a review. Evidence Policy J Res Debate Prac.

[7] Orton L, Lloyd-Williams F, Taylor-Robinson D, O'Flaherty M, Capewell S (2011). The use of research evidence in public health decision making processes: systematic review. PLoS ONE.

[8] Straus SE, Tetroe J, Graham ID, Zwarenstein M, Bhattacharyya O, Shepperd S (2010). Monitoring use of knowledge and evaluating outcomes. Can Med Assoc J.

[9] Rymer L (2011). Measuring The Impact Of Research – The Context For Metric Development.

[10] Oliver K, Innvar S, Lorenc T, Woodman J, Thomas J (2014). A systematic review of barriers to and facilitators of the use of evidence by policymakers. BMC Health Serv Res.

[11] Amara N, Ouimet M, Landry R (2004). New Evidence on Instrumental, Conceptual, and Symbolic Utilization of University Research in Government Agencies. Sci Commun.

[12] Ouimet M, Bédard PO, Turgeon J, Lavis JN, Gélineau F, Gagnon F (2010). Correlates of consulting research evidence among policy analysts in government ministries: A cross-sectional survey. Evidence Policy.

[13] Oxman AD, Lavis JN, Lewin S, Fretheim A (2009). SUPPORT Tools for evidence-informed health Policymaking (STP) 10: Taking equity into consideration when assessing the findings of a systematic review. Health Res Policy Syst.

[14] Landry R, Lamari M, Amara N (2003). The Extent and Determinants of the Utilization of University Research in Government Agencies. Public Adm Rev.

[15] Lavis JN, Ross SE, Hurley JE (2002). Examining the Role of Health Services Research in Public Policymaking. Milbank Q.

[16] Davies P (2012). The State of Evidence-Based Policy Evaluation and its Role in Policy Formation. Natl Inst Econ Rev.

[17] Jansen MW, van Oers HAM, Kok G, de Vries NK. Public health: disconnections between policy, practice and research. J Health Serv Res Policy. 2010;37(8). 10.1186/1478-4505-8-37 PMC3022611 21194428

[18] Ritter A (2009). How do drug policy makers access research evidence?. Int J Drug Policy.

[19] Jewell CJ, Bero LA (2008). “Developing good taste in evidence”: Facilitators of and hindrances to evidence-informed health policymaking in state government. Milbank Q.

[20] Haynes AS, Derrick GE, Redman S, Hall WD, Gillespie JA, Chapman S (2012). Identifying Trustworthy Experts: How Do Policymakers Find and Assess Public Health Researchers Worth Consulting or Collaborating With?. PLoS One.

[21] Higgins JW, Strange K, Scarr J, Pennock M, Barr V, Yew A, et al. “It’s a feel. That’s what a lot of our evidence would consist of”: public health practitioners’ perspectives on evidence. Eval Health Prof. 2011;34(3). 10.1177/0163278710393954 21224264

[22] Dobbins M, Jack S, Thomas H, Kothari A (2007). Public Health Decision-Makers’ Informational Needs and Preferences for Receiving Research Evidence. Worldviews Evid-Based Nurs.

[23] Gray M, Joy E, Plath D, Webb SA (2013). Opinions about evidence: a study of social workers’ attitudes towards evidence-based practice. J Soc Work.

[24] Kothari A, Edward N, Hamel N, Judd M. Is research working for you? validating a tool to examine the capacity of health organizations to use research. Implement Sci. 2009;4(46). 10.1186/1748-5908-4-46 PMC2727486 19627601

[25] Chagnon F, Pouliot L, Malo C, Gervais M-J, Pigeon M-E (2010). Comparison of determinants of research knowledge utilization by practitioners and administrators in the field of child and family social services. Implement Sci.

[26] Australian Bureau of Statistics (2012). Australian Demographic Statistics.

[27] Foundation PIE (2011). Understanding Accident Compensation in Australia and New Zealand.

[28] Michie S, Johnston M, Abraham C, Lawton R, Parker D, Walker A (2005). Making psychological theory useful for implementing evidence based practice: a consensus approach. Quality Safety Health Care.

[29] Campbell DM, Redman S, Jorm L, Cooke M, Zwi AB, Rychetnik L. Increasing the use of evidence in health policy: practice and views of policy makers and researchers. Aust New Zealand Health Policy. 2009;6(21). 10.1186/1743-8462-6-21 PMC2739528 19698186

[30] Mitton C, Adair CE, McKenzie E, Patten SB, Waye PB (2007). Knowledge transfer and exchange: review and synthesis of the literature. Milbank Q.

[31] Gold M (2009). Pathways to the use of health services research in policy. Health Serv Res.

[32] Zardo P, Collie A, Livingstone C (2014). Extenal factors affecting decision-making and use of research in an Australian public health policy environment. Soc Sci Med.

[33] Weiss CH (1979). The many meanings of research utilization. Public Adm Rev.

[34] Beyer JM, Harrison MT (1982). The Utilization Process: A Conceptual Framework and Synthesis of Empirical Findings. Adm Sci Q.

[35] Hanney SR, Gonzalez-Block MA, Buxton MJ, Kogan M. The utilisation of health research in policy-making: concepts, examples and methods of assessment. Health Res Policy Syst. 2003;1(2). 10.1186/1478-4505-1-2 PMC151555 12646071

[36] Martin GP, Currie G, Lockett A (2011). Prospects for knowledge exchange in health policy and management: institutional and epistemic boundaries. J Health Serv Res Policy.

[37] Lavis JN, Robertson D, Woodside JM, McLeod CB, Abelson J, Group TKTS (2003). How Can Research Organizations More Effectively Transfer Research Knowledge to Decision Makers?. Milbank Quarterly.

[38] DeVellis RF (2012). Scale Development Theory and Applications.

[39] Streiner D (2003). Starting at the Beginning: An Introduction to Coefficient Alpha and Internal Consistency. J Pers Assess.

[40] Russell J, Greenhalgh T, Byrne E, McDonnell J (2008). Recognizing rhetoric in health care policy analysis. J Health Serv Res Policy.

[41] Institute for Safety Compensation and Recovery Research (2014). Annual Report: Making a difference through research.

[42] Zardo P, Collie A (2014). Measuring use of research evidence in public health policy: a policy content analysis. BMC Public Health.

[43] Contandriopoulos D, Lemire M, Denis JL, Tremblay E (2010). Knowledge exchange processes in organizations and policy arenas: A narrative systematic review of the literature. Milbank Q.

[44] Greenhalgh T, Robert G, Macfarlane F, Bate P, Kyriakidou O (2004). Diffusion of innovations in service organisations: systematic review and recommendations. Millbank Q.

[45] Pawson R, Greenhalgh T, Harvey G, Walshe K (2005). Realist review - a new method of systematic review designed for complex policy interventions. J Health Serv Res Policy.

[46] Althaus C, Bridgman P, Davis G (2007). The Australian policy handbook.

[47] Colebatch HK (2006). Beyond the policy cycle : the policy process in Australia.

[48] Anderson M (2008). Evaluation of the Executive Training for Research Application (EXTRA) Program: Design and Early Findings. Health Care Policy.

[49] Lewis JM (2005). Health Policy and Politics: networks, ideas and power.

[50] Smith KE, Katikireddi SV (2013). A glossary of theories for understanding policymaking. J Epidemiol Community Health.

[51] Zardo P, Collie A, Livingstone C. Organisational factors affecting policy and programme decision making in a public health policy environment. Evidence & Policy: A Journal of Research, Debate and Practice. 2014;Online(26 November).

[52] Lindblom CE (1959). The Science of “Muddling Through”. Public Adm Rev.

[53] Innvær S, Vist G, Trommald M, Oxman A (2002). Health policy-makers’ perceptions of their use of evidence: a systematic review. J Health Serv Res Policy.

[54] Zardo P, Collie A (2014). Predicting research use in a public health policy environment: results of a logistic regression analysis. Implement Sci.

[55] Kingdon JW (1995). Agendas, alternatives, and public policies. 2nd ed. ed.

[56] Weiss CH, Bucuvalas MJ (1980). Truth Tests and Utility Tests: Decision-Makers’ Frames of Reference for Social Science Research. Am Sociol Rev.

